# In situ formation of nanocrystalline Ni(OH)_2_ in alkaline electrolyte explains superior capacitance and cycling stability of Ni_3_S_2_/NF electrodes

**DOI:** 10.1038/s41598-026-42576-y

**Published:** 2026-03-05

**Authors:** Kh. A. Abdullin, M. T. Gabdullin, L. V. Gritsenko, Zh. K. Kalkozova, Zh. S. Kanatov, A. A. Markhabayeva, R. R. Nemkayeva, D. Zhapargali, M. Mirzaeian

**Affiliations:** 1 National Nanotechnology Laboratory of Open Type of Al-Farabi Kazakh National University, al-Farabi ave., 71, Almaty, 050040 Kazakhstan; 2 School of Materials Science and Green Technology, Kazakh-British Technical University, Tole bi Street, 59, Almaty, 050000 Kazakhstan; 3 General Physics Department, Satbayev University, Satpayev str., 22, Almaty, 050013 Kazakhstan; 4 Department of Materials Science, Nanotechnology and Engineering Physics, Satbayev University, Satpayev str., 22, Almaty, 050013 Kazakhstan; 5 School of Computing, Engineering and Physical Sciences, University of the West of Scotland, Paisley, PA1 2BE UK

**Keywords:** Ni_3_S_2_-based electrode, Hydrothermal route, Capacitance, Nickel foam, Hybrid supercapacitors, Chemistry, Energy science and technology, Materials science, Nanoscience and technology

## Abstract

The free-standing Ni_3_S_2_ electrode was fabricated via a simple one-step hydrothermal method at 160 °C through the sulfidization of nickel foam (NF). The as-synthesized samples exhibited X-ray diffraction (XRD) patterns and Raman spectra consistent with the formation of Ni_3_S_2_ Although the XRD reflections of the Ni_3_S_2_ lattice remained unchanged after short-term electrochemical activation in 3.5 M KOH electrolyte, the Raman spectrum evolved to resemble that of oxidized nickel species, indicating a surface transformation of the electrode under electrochemical conditions. Prolonged redox cycling of the electrode for more than 10,000 cycles resulted in noticeable modifications of the XRD pattern. In addition to the characteristic reflections of the Ni_3_S_2_ phase, the formation of β-Ni(OH)_2_ nanoparticles was observed, with an average crystallite size of approximately 2.8 nm, as estimated using the Scherrer equation. The resulting heterostructured Ni(OH)_2_/Ni_3_S_2_/NF electrode exhibited an areal capacitance of 4.1 C cm^–2^ at a current density of 20 mA cm^–2^ when evaluated in a three-electrode configuration. The electrode demonstrated progressively enhanced capacitance stability with continued cycling. Capacitance retention increased from 19% after the first 10,000 cycles to 41% after the second 10,000 cycles, 66% after the third 8,000 cycles, and ultimately 79% after the fourth 6,000 cycles. Taking into account the electrochemical activation occurring between cycling intervals, the overall capacitance retention reached 72% after more than 30,000 cycles. Although the electrode capacitance decreased during galvanostatic charge–discharge (GCD) cycling, it could be partially recovered through subsequent cyclic voltammetry (CV) activation. Concurrently, a broad XRD reflection attributable to the α-Ni(OH)_2_ phase emerged, providing valuable insight into the structural evolution of the electrode and the underlying electrochemical energy-storage mechanism. The Ni(OH)_2_/Ni_3_S_2_/NF electrode aged through 10,000 GCD cycles was subsequently employed as the positive battery-type electrode in a hybrid supercapacitor, paired with an activated carbon negative electrode. The assembled device, with a Ni_3_S_2_/NF electrode area of 6 cm^2^, delivered an areal capacitance of 4.63 C cm^–2^ at a current density of 4 mA cm^–2^ and maintained 83.5% capacitance retention after more than 20,000 charge–discharge cycles.

**Supplementary Information:**

The online version contains supplementary material available at 10.1038/s41598-026-42576-y.

## Introduction

Renewable energy sources are meeting an increasingly significant share of global energy demand while minimizing environmental impact and conserving natural resources. To further expand the adoption of renewable energy, the development of efficient electrical energy storage systems is essential. Alongside batteries, supercapacitors represent key components of modern electrochemical energy storage technologies. They are designed to store energy efficiently, offering rapid charge–discharge capability and high power density, thereby providing reliable backup power and mitigating transient power surges, which enhances the overall stability and resilience of energy systems^1–5^. Electrochemical supercapacitors employ two main types of electrodes: capacitor-type electrodes, in which charge storage occurs through electrostatic adsorption and fast surface redox reactions, and battery-type electrodes, where energy storage is governed by bulk redox processes involving ion intercalation^6–8^. Battery-type electrodes used in hybrid electrochemical supercapacitors combine high energy density with high power density and long-term cycling stability. These electrodes are commonly fabricated from carbon-based composites, conductive polymers, metal oxides, fluorides, and other transition-metal compounds^5,9–14^. Nickel-based compounds, in particular, have attracted considerable attention for supercapacitor applications^15–17^. Even thin nickel oxide layers formed via in situ oxidation on the surface of nickel foam (NF) have demonstrated strong potential for scalable supercapacitor electrode fabrication^18^.

Nickel sulfides, however, exhibit significantly higher specific capacities than nickel oxides^19^. This advantage is generally attributed to their high theoretical capacity, good electrical conductivity, structural stability of their layered phases, and ability to undergo reversible redox reactions. Owing to these favorable characteristics, nickel sulfides are widely regarded as highly promising electrode materials, as highlighted in numerous studies^19–25^. Nickel sulfides have been extensively investigated as battery-type electrode materials, in part due to the suitability of nickel foam (NF) as a conductive substrate. NF provides a three-dimensional framework with a relatively large specific surface area, high electrical and thermal conductivity, mechanical robustness, and chemical stability in alkaline electrolytes. Nickel sulfide layers can be directly grown on the surface of NF, with the foam simultaneously acting as a source of Ni^2+^ ions during hydrothermal synthesis. This strategy promotes chemical and structural compatibility between the substrate and the active material, ensures strong adhesion of the redox-active layer, and provides both structural integrity and stable electrochemical performance during prolonged cycling, along with low interfacial resistance^22,23^. Moreover, nickel sulfides exhibit high theoretical capacities. Among the various synthesis methods, including hydrothermal synthesis and electrodeposition, the most stable—and consequently the most extensively studied—phase formed on NF is Ni_3_S_2_^26,27^. The Ni_3_S_2_ phase possesses high electrical conductivity, which facilitates rapid electron transport during charge–discharge processes^28^. Nickel atoms actively participate in reversible redox reactions, readily transitioning between Ni^2+^ and Ni^3+^ oxidation states, thereby delivering high pseudocapacitance through bulk Faradaic processes.

Numerous studies have reported exceptionally high capacitance values for Ni_3_S_2_-based electrodes, reaching up to 1649 F g⁻¹^27^ and 3296 F g⁻¹^29^. These experimental values can be compared with the theoretical specific capacity of Ni_3_S_2_, which is 223.16 mAh g⁻¹ (equivalent to 839.4 C g⁻¹) in KOH electrolyte. At a typical operating voltage window of ΔV = 0.5 V, this corresponds to a theoretical specific capacitance of 1678.8 F g⁻¹. In addition, many studies have highlighted the excellent cycling stability of Ni_3_S_2_-based electrodes. For example, reports such as^30–38^, in which Ni_3_S_2_/NF structures were synthesized via sulfidation of nickel foam using sulfur-containing precursors, observed only a slight decrease—or in some cases a modest increase—in capacitance during prolonged cycling.

Excellent cycling performance has also been reported for Ni_3_S_2_/NF electrodes modified with an additional Ni(OH)_2_ layer grown through a secondary hydrothermal process. For instance, in^39^, Ni(OH)_2_ nanosheets derived from a metal–organic framework (MOF) precursor were deposited onto the Ni_3_S_2_/NF surface via a second hydrothermal step followed by alkaline treatment. After 500 cyclic voltammetry (CV) cycles, the electrode retained 90.4% of its initial specific capacitance. Similarly, in^40^, a mixed Ni–Co hydroxide layer was introduced during the second stage of hydrothermal synthesis. The resulting composite electrode delivered a capacitance of 2694 F g⁻¹ at a current density of 2 A g⁻¹ and demonstrated outstanding cycling stability, retaining 94% of its initial capacitance after 15,000 cycles.

Nickel hydroxide (Ni(OH)_2_) has long been recognized as a promising supercapacitor material due to its high electrochemical performance and excellent cycling stability. Accordingly, Ni(OH)_2_/NF architectures fabricated on nickel foam substrates have been extensively investigated as supercapacitor electrodes^41–43^. For example, Ref^44^. reported a high areal specific capacity of 3.68 mAh cm^–2^ (13.2 C cm^–2^), along with a remarkable increase in capacitance to 116% of the initial value after 2000 charge–discharge cycles. In Ref^45^., controlled growth and in situ phase transformation from β-Ni(OH)_2_ to nitrated α-Ni(OH)_2_ (i.e., Ni_3_(NO_3_)_2_(OH)_4_) on nickel foam were successfully achieved via a conventional mild hydrothermal process by adjusting reagent concentrations. Electrochemical characterization revealed that the α-type nickel hydroxide electrode exhibited a significantly higher specific capacity than its β-type counterpart. Specifically, the α-phase delivered a gravimetric capacitance of 2075 F g⁻¹ at a current density of 0.5 A g⁻¹, approaching its theoretical limit.

Composite Ni(OH)_2_/Ni_3_S_2_/NF electrodes have also been explored and were found to exhibit high specific capacities. For instance, the synthesis of α-Ni(OH)_2_ nanosheets grown on a nickel foam substrate followed by sulfidation resulted in a capacitance of up to 2885 F g⁻¹ at a current density of 2 A g⁻¹^46^. In Ref^47^., a Ni(OH)_2_ layer was formed during hydrothermal synthesis at 180 °C with varying reaction times, leading to the formation of Ni_3_S_2_@Ni(OH)_2_/3D graphene network (3DGN) architectures. These structures exhibited significantly higher capacitance than those lacking either the Ni(OH)_2_ or Ni_3_S_2_ component. The enhanced electrochemical performance was attributed to a synergistic effect between the abundant electrochemically active sites provided by the Ni(OH)_2_ nanosheets and the rapid electron transport enabled by the Ni_3_S_2_ layer.

In Ref^48^., a Ni foam– Ni_3_S_2_@Ni(OH)_2_–graphene sandwich structure was fabricated using a two-step synthesis route. In the first step, a Ni_3_S_2_ layer was formed on nickel foam via hydrothermal treatment in a thioacetamide-containing medium. In the second step, graphene oxide was deposited hydrothermally while the Ni_3_S_2_ surface underwent partial hydrolysis to form Ni(OH)_2_. The outstanding electrochemical performance of the resulting NF– Ni_3_S_2_@Ni(OH)_2_–graphene electrode was attributed to its unique hierarchical microstructure, which provided a high density of active sites from Ni(OH)_2_ nanosheets or nanoflowers, rapid electron transport through the nanocrystalline Ni_3_S_2_ layer, and efficient electron collection by both graphene and the nickel foam substrate. The slight increase in capacitance observed during cycling was ascribed to an activation process, during which improved surface wetting via ion intercalation and deintercalation increased the number of electrochemically active sites.

It should be noted that Ni(OH)_2_ layers can form in situ on the surface of Ni_3_S_2_ during electrochemical cycling in alkaline electrolytes with high KOH concentrations. Under anodic polarization, nickel sulfide (Ni_3_S_2_) readily undergoes surface oxidation, leading to the formation of Ni(OH)_2_ in the near-surface region. Consequently, during electrochemical testing of Ni_3_S_2_/NF electrodes in alkaline media, Ni(OH)_2_/Ni_3_S_2_/NF composite structures inevitably develop. Despite its significance, this phenomenon is often overlooked, and to the best of our knowledge, only one study^49^ has explicitly addressed the influence of in situ–formed Ni(OH)_2_ layers on the capacitance of Ni_3_S_2_-based electrodes. In that study, a Ni_3_S_2_ layer on nickel foam was prepared by annealing the substrate in an H₂S atmosphere (5% H₂S–95% N₂) at 400 °C for 60 min. Electrochemical testing in 2 M KOH electrolyte revealed that, during charge–discharge cycling, the effective surface area increased by more than two orders of magnitude, while the areal capacitance rose to 9.88 F cm^–2^ at a current density of 10 mA cm^–2^—nearly 90 times higher than the initial value of 112.5 mF cm^–2^. SEM elemental mapping indicated a predominance of Ni and O in the near-surface region, with sulfur contents limited to 4.8–5.8%, suggesting substantial surface restructuring. The formation of the Ni(OH)_2_ phase was further confirmed by the appearance of broad Raman bands at 460 and 536 cm^–1^, whereas no Raman features corresponding to Ni_3_S_2_ were detected. These results indicate that the exceptionally high capacitance originates from the in situ formation of porous Ni(OH)_2_ layers on the Ni_3_S_2_ surface.

Given that many studies on nickel sulfide electrodes employ alkaline electrolytes, it is essential to account for the formation of a hydroxide surface layer induced by electrochemical reactions. This layer can exert a decisive influence on the electrochemical properties of Ni_3_S_2_/NF electrodes, which are widely regarded as promising candidates for supercapacitor applications^46,50,51^.

Our results demonstrate that the high capacitance of hydrothermally synthesized Ni_3_S_2_/NF electrodes, their excellent cycling stability, and the partial recovery of capacitance after prolonged cycling are primarily attributable to the in situ formation of an oxidized surface layer. Notably, even at early stages of cycling (after approximately 30 cycles), the Raman bands characteristic of the Ni_3_S_2_ phase disappear, while new bands associated with nickel oxide species emerge. Continued electrochemical cycling leads to the development of a stable Raman spectrum characteristic of the bending and stretching vibration modes of Ni(III)–O bonds in NiOOH. As the number of electrochemical cycles increases to 10,000–20,000, oxide phases become detectable in the XRD patterns, revealing the formation of a nanocrystalline β-Ni(OH)_2_ phase, which partially transforms into the α-Ni(OH)_2_ phase upon further electrochemical activation. The evolution of this oxidized surface layer is accompanied by a pronounced synergistic effect, resulting in a significant enhancement of the overall electrochemical performance.

## Materials and methods

Analytical grade Ni(CH_3_COO)_2_·4 H₂O nickel acetate, CH_4_N_2_S thiourea, PTFE dispersion 60 wt% in H_2_O (Sigma Aldrich, St. Louis, MO, USA), activated carbon (Fuzhou Yihuan Carbon Co., Fuzhou City, China) were used as received. Nickel foam with a thickness of 1.5 mm (Shenzhen Tianchenghe Technology Co. Ltd.) was used as the substrate. It was cleaned via sonication with 1 M HCl solution before hydrothermal synthesis. MilliQ water (18.2 Mohm×cm) was obtained by Water Purification System AQUAMAX—Ultra 370 Series (YL Instrument Co., Anyang, Korea).

A one-pot hydrothermal method was employed to synthesize Ni_3_S_2_ layers on nickel substrates with a specific loading of 0.03 g cm^–2^. For this purpose, two precursor solutions were prepared: 0.05 M nickel acetate and 0.1 M thiourea. Immediately prior to synthesis, 30 mL of each solution was added to a fluoroplastic beaker containing a pre-cleaned nickel foam (NF) substrate with dimensions of 1 cm×6 cm. The PTFE beaker was then sealed in a metal autoclave and placed in a muffle furnace preheated to 160 °C. Preliminary measurements of the autoclave heating showed that the temperature of the outer steel surface reached 155 °C after 3 h in the muffle furnace. The total synthesis time was 3.5 h. Following synthesis, the autoclave was removed from the furnace and allowed to cool in air. Once cooled to room temperature, the NF sample was taken out of the PTFE container, rinsed with water, treated in an ultrasonic bath for 30 min, and then dried.

The morphology of the samples was examined using a Quanta 200i scanning electron microscope (FEI). The phase composition was analyzed with a MiniFlex diffractometer (Rigaku) employing Cu Kα radiation (λ = 1.5418 Å). Raman spectra, excited at a wavelength of 473 nm, were recorded with an NTEGRA spectrometer (NT-MDT, Zelenograd, Russia). XPS spectra were obtained using a NEXSA X-ray photoelectron spectrometer (Thermo Scientific, Waltham, MA, USA). The electrochemical properties of the electrodes were evaluated using a CorrTest CS2350 bipotentiostat (Wuhan Corrtest Instruments Corp., Ltd., Wuhan, China) through cyclic voltammetry (CV), galvanostatic charge–discharge (GCD), and electrochemical impedance spectroscopy (EIS) measurements.

The capacitance of the electrodes was calculated from CV and GCD measurements using Eqs. (1) and (2), respectively,

1 $$\mathrm{C}=\frac{1}{2\nu({U}_{max}-{U}_{min})}\oint I\left(V\right)dV,$$

2 $$\mathrm{C}=\frac{I\varDelta t}{{\Delta}U},$$

where *v* (V s^–1^) is the scan rate and U_max_–U_min_ (V) is the potential window in the CV method. The current *I* (A) was integrated over a complete CV cycle. For GCD measurements, the capacitance was determined from the galvanostatic discharge current *I* (A), discharge time Δ*t* (s), and voltage drop Δ*U* (V), considered as more reliable parameters for evaluating electrode capacitance. To determine the specific capacity, the mass of NF samples was measured before and after the synthesis. The areal density of the untreated NF was 0.0314 g cm^–2^. On average, the mass increased by 4% (1.2 mg cm^–2^) under the selected synthesis conditions. Assuming that Ni_3_S_2_ formed through nickel sulfidation, the mass of the synthesized Ni_3_S_2_ on the NF substrate was estimated to be 4.5 mg cm^–2^. The electrochemical performance of the Ni_3_S_2_/NF electrodes were investigated in a three-electrode system employing a large-area carbon counter electrode and an Ag/AgCl reference electrode. The asymmetric capacitor was tested in a two-electrode configuration, with the Ni_3_S_2_/NF electrode serving as the positive electrode and an activated carbon (AC) electrode as the negative electrode. The AC electrode was fabricated by the conventional method, using an aqueous slurry of activated carbon, carbon black, and PTFE binder in a weight ratio of 8:1:1. The slurry was rolled into a film approximately 100 μm thick, which was then pressed into nickel foam and dried.

## Results

**Fig. 1:**
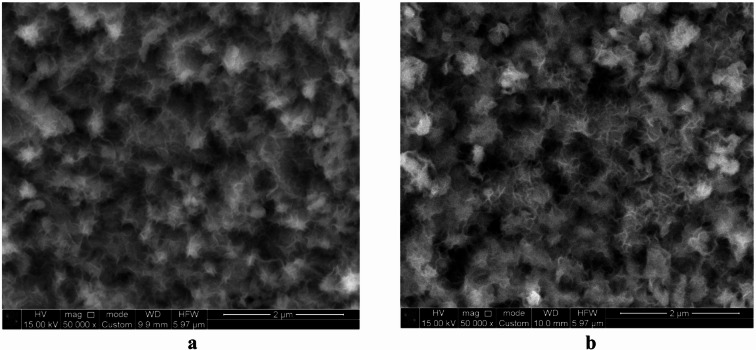
SEM images of the surface of NF substrates immediately after synthesis **(a)** and after CV measurements **(b)**.

SEM images of the substrates obtained immediately after the synthesis of the Ni_3_S_2_ layers (Fig. 1a) reveal the formation of porous, nanosheet-like structures on the surface of the nickel foam. This morphology is characteristic of Ni_3_S_2_ synthesized via hydrothermal methods on NF substrates^27,35^.

**Fig. 2:**
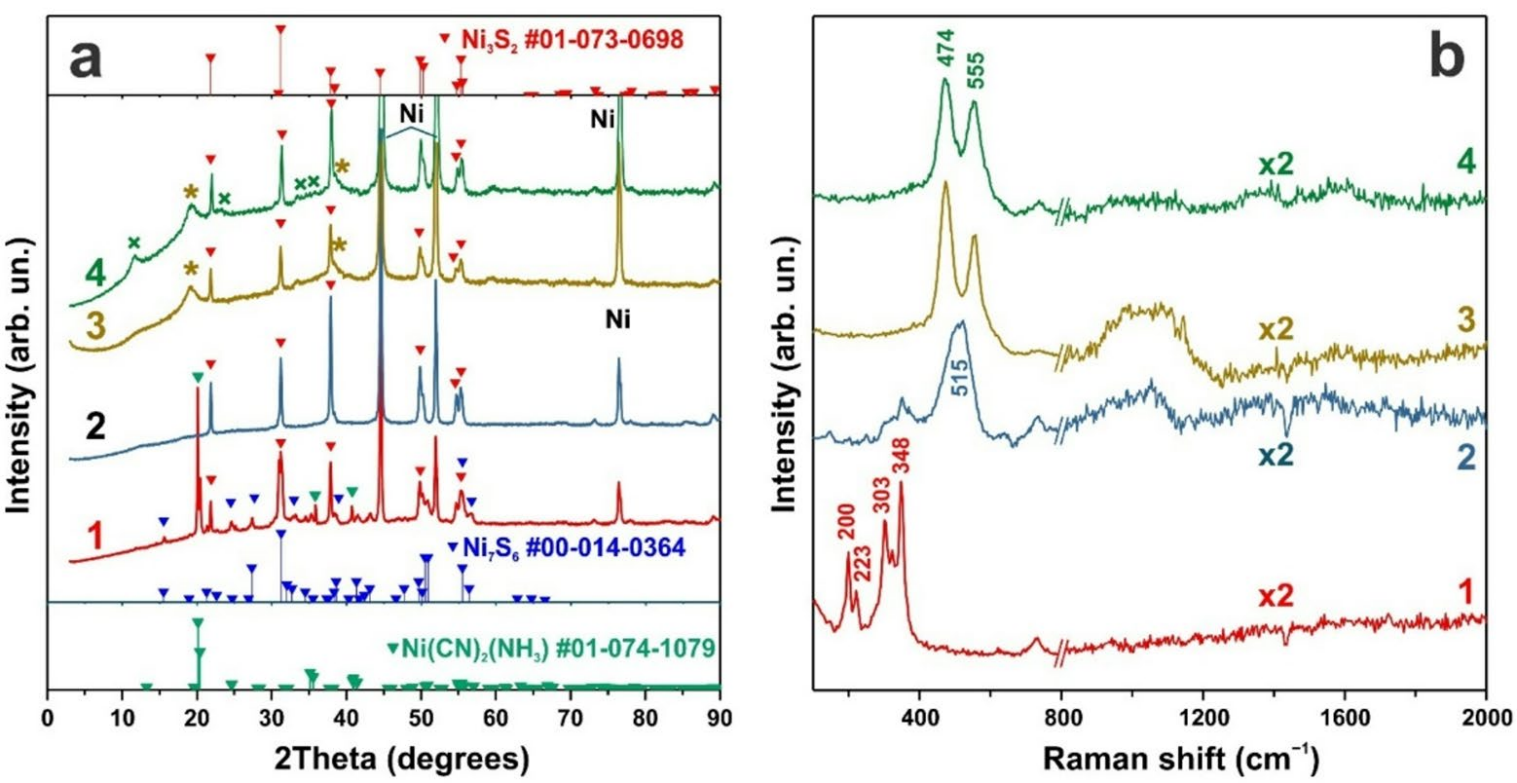
XRD patterns **(a)** and Raman spectra **(b)** of the sample in different states: as-synthesized (curve 1), after 80 cyclic voltammetry (CV) cycles (curve 2), after 10,000 galvanostatic charge–discharge (GCD) cycles (curve 3), and after electrochemical activation during subsequent CV cycling (curve 4). The XRD reflections corresponding to β-NiOOH are marked with an asterisk (PDF card No. 00–006−0141), while the reflection of α−3Ni(OH)_2_·2H_2_O is indicated by a cross (PDF card No. 00–022−0444).

The XRD patterns of the as-synthesized layer indicate that Ni_3_S_2_ is the predominant phase, accompanied by the presence of a Ni(CN)_2_(NH_3_) complex (Fig. 2a, curve 1). Because the decomposition of thiourea releases cyanide (CN^–^) and ammonia (NH_3_), the formation of this complex compound is highly plausible. The diffraction peaks associated with Ni(CN)_2_(NH_3_) disappear rapidly after several cyclic voltammetry (CV) cycles (Fig. 2a, curve 2), leaving only reflections attributable to the Ni_3_S_2_ phase. A slight modification of the electrode structure, manifested as increased porosity (Fig. 1b), is presumably associated with the removal of the complex compound during electrochemical cycling and the initial stages of surface oxidation.

Raman spectroscopy further confirms the presence of the Ni_3_S_2_ phase after synthesis, with its characteristic vibrational features clearly observed. The Raman spectrum of the as-synthesized sample exhibits five bands at 348, 323, 303, 223, and 200 cm^–1^ (Fig. 2b, curve 1), which can be assigned to the E(1), A_1_(1), E(2), E(3), and E(4) Raman modes, respectively, in good agreement with calculated Raman spectra of Ni_3_S_2_^52^. The poorly resolved feature on the low-frequency side of the 200 cm^–1^ band can be attributed to the A_1_(2) mode, which is typically observed near 188 cm^–1^ and is also characteristic of Ni_3_S_2_. Similar Raman spectra were observed in NiMn_2_S_4_^53^. The narrow linewidths of the observed Ni_3_S_2_ Raman bands, together with the absence of additional peaks corresponding to NiS, Ni_3_S_4_, or NiS_2_, confirm the high phase purity of the synthesized samples.

**Fig. 3:**
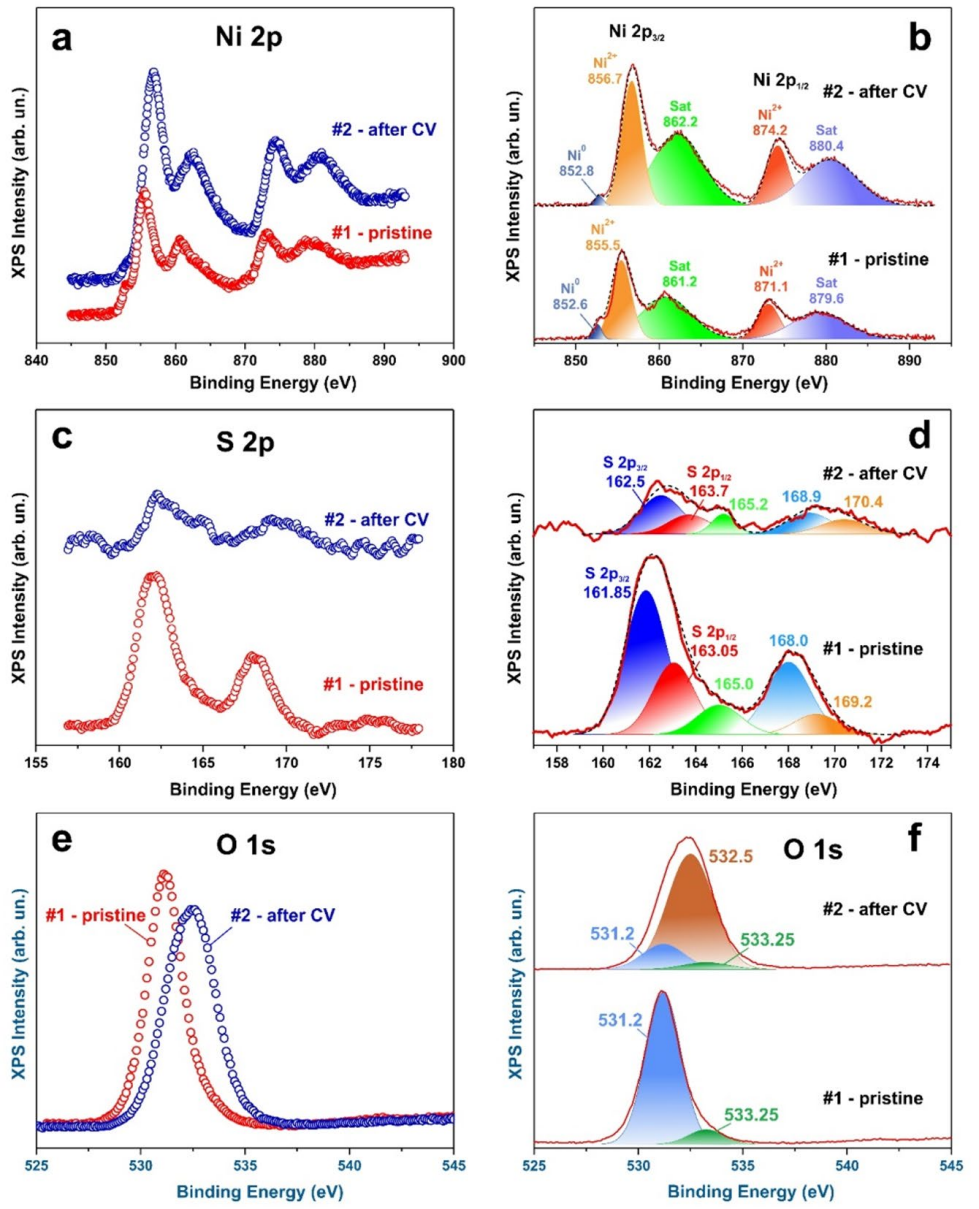
High-resolution XPS spectra of Ni **(a**,** b)**, S **(c**,** d)**, and O **(e**,** f)** acquired before (curve #1) and after cyclic voltammetry (CV) cycling (curve #2), with Gaussian deconvolution shown in panels (c), (d), and (f).

To probe the near-surface chemical states of the synthesized electrodes, X-ray photoelectron spectroscopy (XPS) was performed (Fig. 3). The Ni 2p spectrum exhibits a peak at 852.6 eV corresponding to metallic Ni^0^, along with a Ni 2p_3/2_ peak at 855.5 eV arising from overlapping contributions of Ni^2+^ and Ni^3+^ species, and a satellite feature at 861.2 eV. The Ni 2p_1/2_ components appear at 871.1 and 879.6 eV, consistent with the characteristic spin–orbit splitting observed for nickel compounds^54^.

The S 2p spectrum is dominated by the main sulfide doublet at approximately 162 eV, comprising S 2p_3/2_ at 161.85 eV and S 2p_1/2_ at 163.05 eV, with a characteristic splitting of 1.2 eV. An additional component at approximately 168 eV, corresponding to an S 2p_3/2_–S 2p_1/2_ doublet, can be attributed to highly oxidized sulfur species at the surface, such as SO₃^2–^ and SO₄^2–^. A minor contribution at approximately 165 eV, observed in the as-synthesized samples, is assigned to neutral sulfur species or organic sulfur compounds present on the surface^55,56^.

The O 1 s spectrum of the as-synthesized samples consists primarily of a band centered at 531.2 eV, which is associated with surface oxidation of Ni_3_S_2_ and may be attributed to O^2–^ species in Ni–O bonds. A peak at 532.5 eV, corresponding to oxygen in hydroxide groups, is absent in the initial samples, while the feature at 533.25 eV is assigned to adsorbed water.

**Fig. 4:**
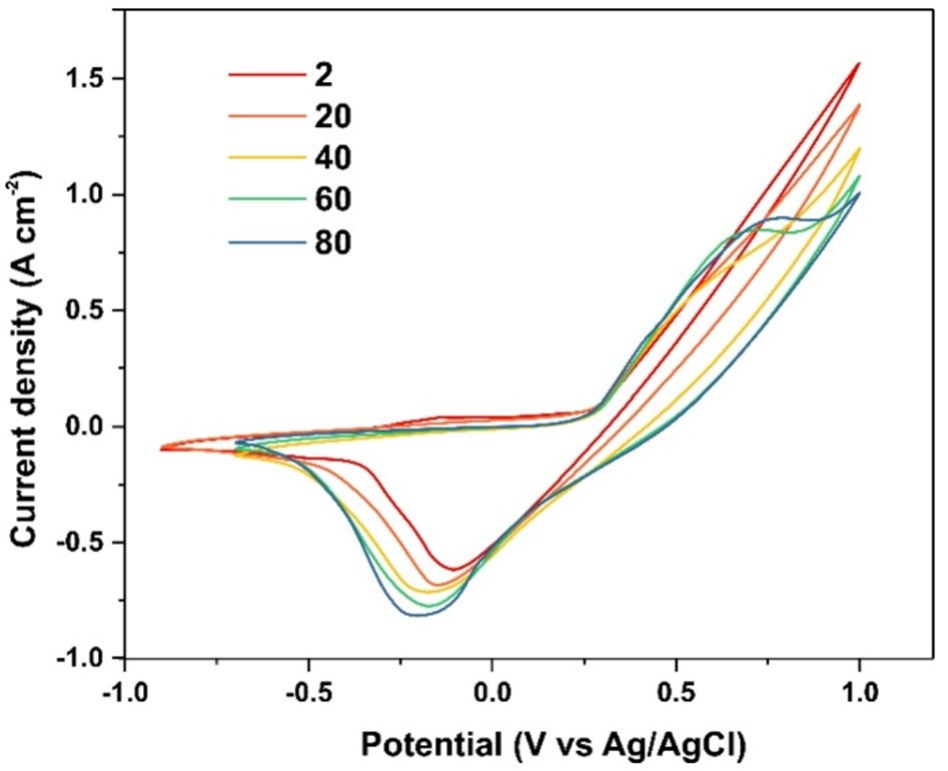
Evolution of the cyclic voltammetry (CV) curves of the as-synthesized Ni_3_S_2_/NF electrode over 80 cycles at a scan rate of 0.1 V s^–1^. The numbers on the curves indicate the corresponding cycle numbers.

Figure 4 illustrates the evolution of the cyclic voltammetry (CV) characteristics of a pristine Ni_3_S_2_/NF electrode with an area of 1 cm^2^, measured in 3.5 M KOH within a potential window of − 0.8 to + 1.0 V vs. Ag/AgCl. During electrochemical cycling, the initial capacitance of the electrode gradually increases, and the CV curves stabilize after approximately 80–100 cycles.

Once the CV response had stabilized, XRD patterns and Raman spectra of the electrode were recorded. The corresponding results are presented in Fig. 2a and b (curve 2). The XRD patterns show the disappearance of reflections associated with the Ni(CN)_2_(NH_3_) phase, leaving only diffraction peaks attributable to the Ni_3_S_2_ phase. In contrast, pronounced changes are observed in the Raman spectra. As shown in Fig. 2b (curve 2), the intensities of the vibrational bands associated with the Ni_3_S_2_ lattice decrease sharply after 80 CV cycles, while a new band emerges at 515 cm^–1^, which can be attributed to Ni–O vibrational modes^57^. Considering that the band gap of Ni_3_S_2_ is approximately 1 eV and that its typical interband absorption coefficient is α ≈ 10^5^ cm⁻¹, excitation at 407 nm results in a penetration depth of approximately 100 nm. Consequently, Raman spectroscopy predominantly probes the near-surface region of the electrode. These observations indicate that the Ni_3_S_2_ phase undergoes structural rearrangement and electrooxidation at an early stage of electrochemical cycling. Such surface-layer transformations of the Ni_3_S_2_/NF electrode account for the observed increase in capacitance during CV cycling.

**Fig. 5:**
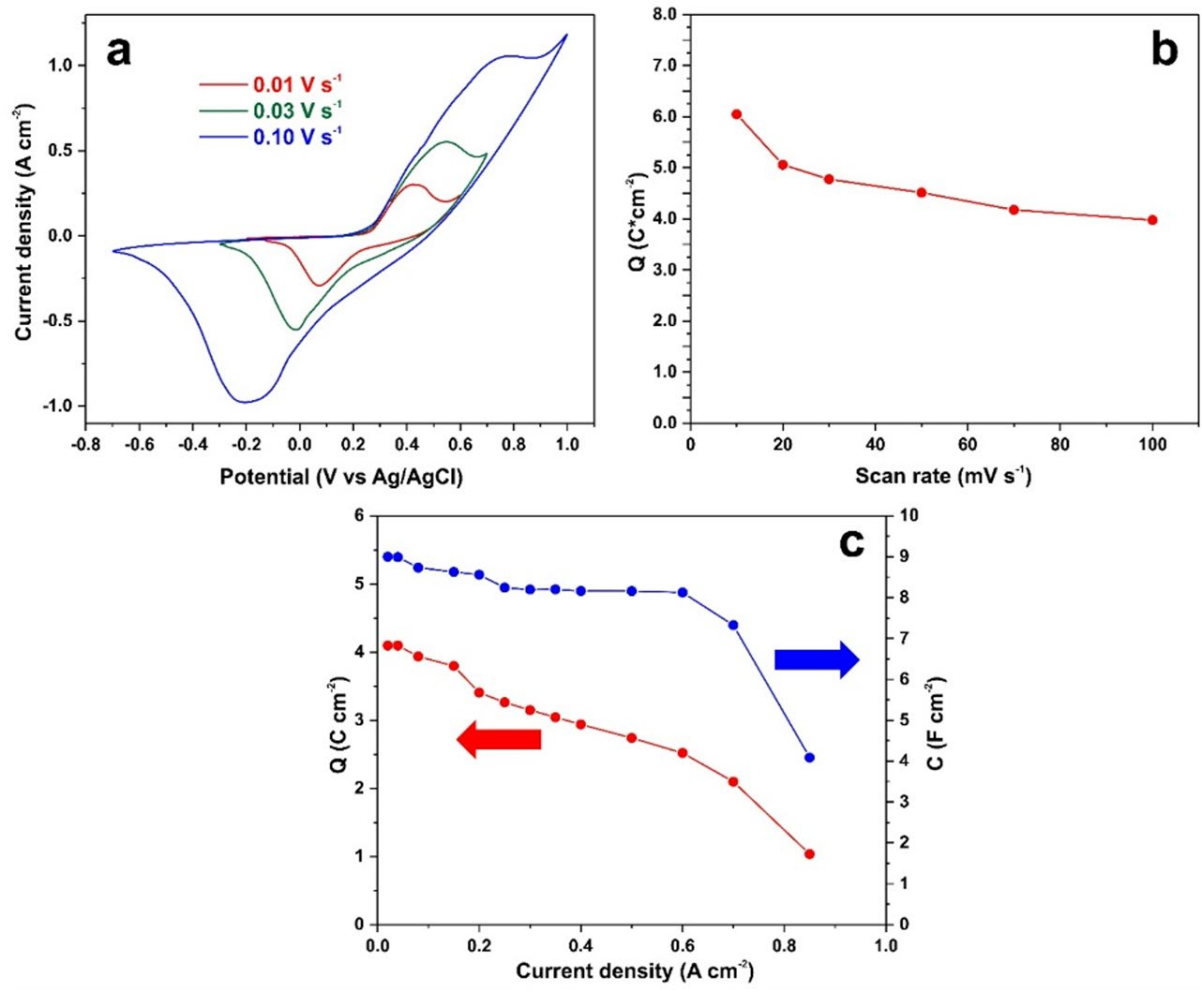
CV curves of the Ni_3_S_2_/NF electrode (S = 1 cm^2^) at different scan rate **(a)**, the electrode capacitance vs. CV scan rate **(b)** and GCD current density **(c)**.

The electrochemical performance of the electrode was evaluated after stabilization of its characteristics, and the corresponding CV results are presented in Fig. 5a. The electrode exhibited a high areal capacitance of up to 6.04 C cm^–2^, with only a gradual decrease in capacitance observed as the discharge rate increased. The ability to maintain electrochemical performance under varying operating conditions and at high current densities is an important practical characteristic of supercapacitor devices. As shown in Fig. 5b, the areal capacitance decreased from 6.04 C cm^–2^ to 3.98 C cm^–2^ as the CV scan rate increased from 10 to 100 mV s^–1^. Similarly, increasing the GCD current density from 20 mA cm^–2^ to more than 800 mA cm^–2^ resulted in a reduction of the areal capacitance from 4.10 C cm^–2^ to 1.00 C cm^–2^ (Fig. 5c). Noticeable attenuation of capacitance was observed only at higher current densities. This behavior can be attributed to the low series resistance of the electrode (Figure S1). At elevated currents, the series resistance causes a reduction in the effective operating voltage across the electrode, which in turn leads to a decrease in the measured capacitance. In addition, kinetic limitations associated with Faradaic (redox) reactions further contribute to the reduction in specific capacitance at high current densities.

**Fig. 6:**
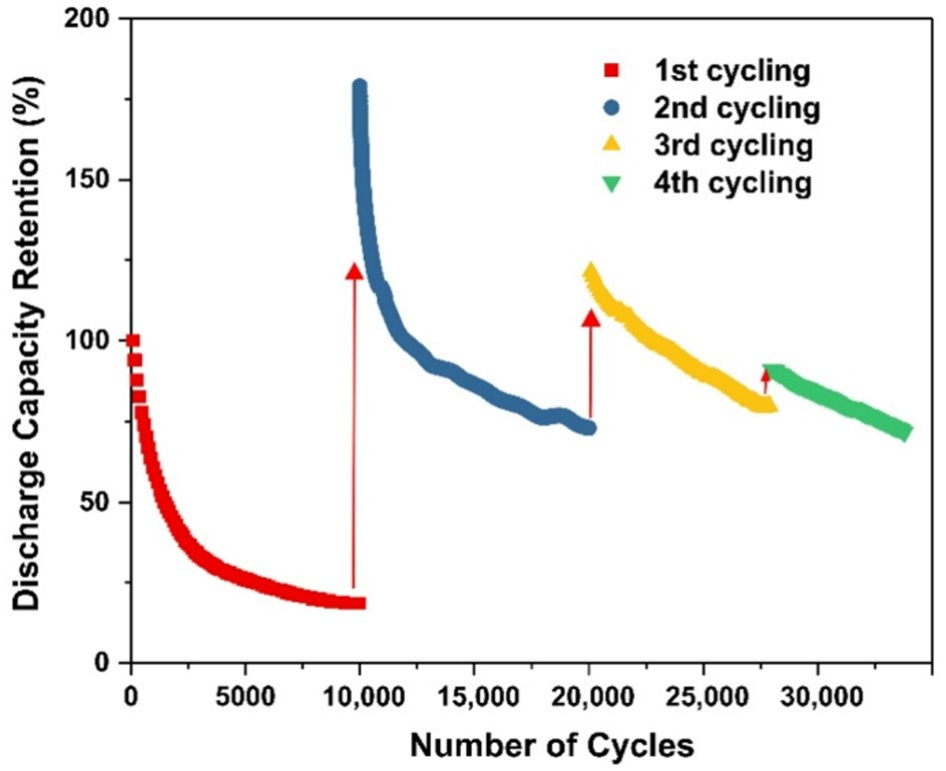
Capacitance retention during four cycling processes at GCD current density of 350 mA cm^–2^ in the range 0–0.45 V vs. Ag/AgCl.

When evaluating the cycling stability of the electrodes under galvanostatic charge–discharge (GCD) conditions, an unusual electrochemical behavior was observed. Figure 6 shows the dependence of capacitance retention on the number of charge–discharge cycles measured in the potential range of 0–0.45 V vs. Ag/AgCl at a current density of 300 mA cm^–2^ over four consecutive cycling stages. As shown, the capacitance decreases sharply during the first cycling stage, retaining only approximately 20% of its initial value after 10,000 GCD cycles. Remarkably, during subsequent cyclic voltammetry (CV) cycling, the capacitance is rapidly and efficiently restored, indicating that surface reactivation and structural rearrangements of the electrode material play a crucial role in recovering electrochemical performance. As illustrated in Figure S2, the electrode capacitance determined by CV within the same potential window as in Fig. 5a exhibits a substantial decrease after 10,000 GCD cycles (curve 1). Nevertheless, a pronounced increase in capacitance is observed upon widening the potential window. Moreover, the capacitance recovery process stabilizes rapidly.

**Fig. 7:**
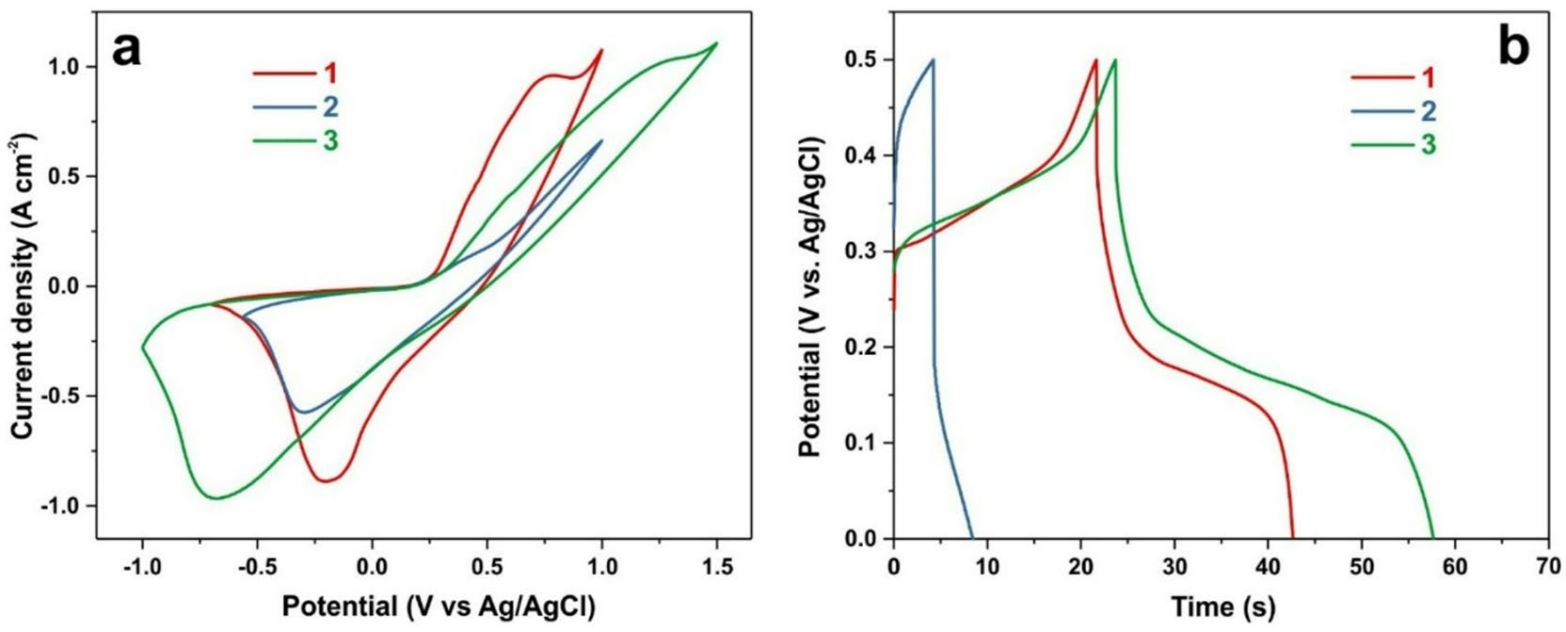
CV **(a)** and GCD **(b)** curves of the Ni_3_S_2_/NF electrode: before GCD cycling (curve 1), after 10,000 GCD cycles (curve 2), and after recovery by 80 CV cycles (curve 3). CV scan rate: 0.1 V s^–1^ (a); GCD discharge current: 0.15 A cm^–2^ (b).

Figure 7a and b compare the CV curves and GCD profiles of the electrode before cycling (curves 1), after 10,000 charge–discharge cycles (curves 2), and after activation by 80 CV cycles (curves 3). The results demonstrate that the capacitance is restored to values even higher than those observed prior to the initial cycling, while the operating potential window is simultaneously expanded. Electrochemical impedance spectroscopy (EIS) measurements (Figure S1) reveal that, after 10,000 GCD cycles followed by CV activation, the series resistance increases by approximately 9%, whereas the capacitance at 0.1 Hz increases by 29%.

As illustrated in Fig. 6, repeated cycling again leads to a decrease in capacitance, while subsequent activation partially restores the electrode performance. However, with successive cycling and activation steps, both the magnitude of capacitance loss and the extent of recovery gradually diminish.

This anomalous behavior is likely associated with structural transformations of the as-prepared Ni_3_S_2_/NF electrode material. To elucidate these changes, Brunauer–Emmett–Teller (BET) surface area measurements, XRD), Raman spectroscopy, and SEM-based elemental mapping were performed and compared for samples before and after electrochemical cycling.

Cycling followed by CV activation was accompanied by an increase in capacitance. To clarify the origin of this enhancement, the specific surface area of the electrode was determined using the BET method. It should be noted that the BET method is particularly effective for powders and porous materials; however, it is fundamentally poorly suited for thin films deposited on massive substrates. In the present case of Ni_3_S_2_ films on nickel foam (NF), gas adsorption occurs on both the film and the substrate, resulting in the measurement of a combined “film + substrate” BET surface area. Moreover, the surface area of the NF substrate can be comparable to that of the nanostructured film, while the mass of the substrate is significantly greater than that of the film itself. Consequently, the contribution of the film cannot be reliably isolated. In addition, the BET method assumes a homogeneous surface, multilayer adsorption, and isotropic adsorption sites. In contrast, thin films typically exhibit surface anisotropy, preferred crystallographic orientation, and significant contributions from grain boundaries and defects. As a result, the BET-derived parameters for such films are physically ambiguous and should be regarded only as approximate estimates.

For the BET measurements, a Ni_3_S_2_/NF electrode was divided into two halves: one half served as the uncycled reference sample, while the other was subjected to 30,000 GCD cycles. The BET-specific surface area was measured to be 0.30 m^2^ g^–1^ for the pristine Ni_3_S_2_/NF electrode and 0.022 m^2^ g^–1^ for the cycled sample. Therefore, considering the limitations of the BET method in the present case, it can be concluded that the physical specific surface area of the electrode decreased after cycling. Consequently, the observed increase in electrochemical capacitance during cycling primarily originates from an enhanced Faradaic contribution, whereas the contribution of the electric double layer remains relatively minor.

The XRD pattern of the electrode after more than 30,000 GCD cycles is shown in Fig. 2a (curve 3), where Ni_3_S_2_ remains the dominant crystalline phase. However, additional reflections appear at 2θ = 19.15° and 38.3° (marked with asterisks), corresponding to the formation of a nanocrystalline β-NiOOH phase (PDF card No. 00–006−0141). The pronounced broadening of the reflection at 19.15° (FWHM = 2.9°) yields an estimated crystallite size of approximately 2.8 nm, as calculated using the Scherrer equation. Following electrochemical activation, new reflections emerge at 2θ = 11.7° and 23.6°, which can be assigned to the α-NiOOH phase (PDF card No. 00–022−0444). The reflection at 11.7° exhibits a FWHM of 1.7°, corresponding to an estimated crystallite size of approximately 4.8 nm.

Raman spectroscopy further confirms oxidation of the Ni_3_S_2_/NF electrode surface accompanied by the formation of NiOOH species. The Raman bands characteristic of the Ni_3_S_2_ phase disappear after approximately 50 CV cycles (Fig. 2b, curve 3). After prolonged GCD cycling, new vibrational modes emerge at 474 and 555 cm^–1^, which are attributed to oxidized nickel species. The two dominant Raman peaks are typically assigned to lattice vibrations of NiOOH: the E_g_ mode (≈ 480 cm^–1^), associated with Ni³⁺–O bending, and the A_1g_ mode (≈ 560 cm^–1^), associated with Ni^3+^–O stretching^57–60^. Such NiOOH-related peaks are generally observed during in situ Raman measurements in alkaline media under positive bias. However, in Ref^59^., these features were also detected *ex situ* in air several hours after cyclic voltammetry (CV) and galvanostatic charge–discharge (GCD) cycling. Although these peaks typically diminish upon storage in air, their decay can, in some cases, be slowed by the formation of protective surface films, such as K_2_CO_3_^57^. In the present study, the NiOOH-related Raman peaks remained observable one day after CV cycling, even after washing the samples to remove electrolyte residues—an unusual observation that warrants further investigation. In addition, a broad Raman band in the 850–1250 cm^–1^ region is attributed to so-called “active oxygen” species within the oxyhydroxide structure^60^. Raman scattering peaks associated with Ni^2+^–O vibrations in Ni(OH)_2_, which are typically expected in the 445–500 cm^–1^ range, may be absent due to the intrinsically low Raman cross section of Ni(OH)_2_^61^.

Following electrochemical exposure of the samples to an alkaline medium, the X-ray photoelectron spectroscopy (XPS) spectra exhibit changes indicative of surface oxidation. In the Ni 2p spectrum, the peak at 852.6 eV corresponding to metallic Ni^0^ decreases markedly in intensity as a result of electrochemical oxidation of the surface (Fig. 3). Moreover, the Ni 2p_3/2_ and Ni 2p_1/2_ peaks shift toward higher binding energies, reflecting the increased electrostatic attraction experienced by electrons in oxidized nickel species. This behavior is consistent with the XRD and Raman results, which indicate the predominance of Ni^2+^ in Ni(OH)_2_ and Ni^3+^ in NiOOH, in contrast to the initial Ni_3_S_2_ phase, where the average nickel oxidation state is lower than + 2.

The intensity of the S 2p signal is markedly reduced after electrochemical cycling (Fig. 3). Possible causes for this decrease include changes in surface morphology, the formation of a resistive surface layer, and a reduction in sulfur concentration in the subsurface region. The first two factors would be expected to affect the XPS intensities of other elements as well. However, the oxygen signal decreases only slightly (Fig. 3e, f), while the nickel signal even increases (Fig. 3a, b). Therefore, the reduction in sulfur intensity is most reasonably attributed to a decreased concentration of sulfur atoms in the subsurface layer. The enhanced relative contribution of the component at 165.2 eV can be assigned to the formation of organic sulfur species or elemental sulfur on the surface during electrochemical oxidation of the electrode. In the O 1 s spectrum, the dominant contribution after cycling arises from oxygen in hydroxide groups at 532.5 eV.

Figures S3 and S4 present SEM-based elemental mapping results, which unequivocally confirm a decrease in sulfur content after cycling. The small thickness of the Ni_3_S_2_ film on the nickel foam substrate (≈ 5 μm) precluded accurate determination of the film composition from surface analysis alone because of interference from the underlying nickel substrate, even at low accelerating voltages. Therefore, elemental composition measurements were performed in a cross-sectional configuration. In the pristine sample (Figure S3), the atomic sulfur-to-nickel ratio was approximately 1:4, which closely matches the stoichiometry of Ni_3_S_2_. The slight excess nickel signal is most likely attributable to contributions from the NF substrate. In contrast, the sample subjected to prolonged GCD cycling (Figure S4) exhibits a pronounced decrease in the atomic sulfur concentration, in full agreement with the XRD, XPS, and Raman spectroscopy results.

## Capacitor testing

To evaluate the practical applicability and stability of the Ni_3_S_2_/NF electrode in an asymmetric supercapacitor, electrochemical tests were conducted in a two-electrode configuration. A Ni_3_S_2_/NF electrode with an active area of 6 cm^2^ was fabricated. Because our results indicated that the electrode properties evolve significantly during electrochemical cycling, the electrode was pre-aged by subjecting it to 10,000 galvanostatic charge–discharge (GCD) cycles in a three-electrode configuration. Given the pronounced nonlinearity of the electrochemical characteristics of the Ni_3_S_2_/NF electrode—and, to a lesser extent, of the AC/NF electrode—the capacitance of the negative activated carbon (AC) electrode was optimized experimentally. This optimization was achieved by maximizing the overall capacitance of the device in a two-electrode configuration, using the Ni_3_S_2_/NF electrode as the positive electrode and a large-area AC/NF electrode as the negative electrode.

Capacitance was determined using the GCD method at a charge–discharge current of 150 mA. To maximize the discharge time, the area of the AC/NF electrode was varied while maintaining a constant area for the Ni_3_S_2_/NF electrode. The electrochemical characteristics of the optimized asymmetric supercapacitor are presented in Figures S5–S8. Cyclic voltammetry (CV) curves recorded at different scan rates, CV profiles as a function of device voltage, and GCD plots of discharge current versus voltage demonstrate high linearity of the device response and excellent rate capability.

**Fig. 8:**
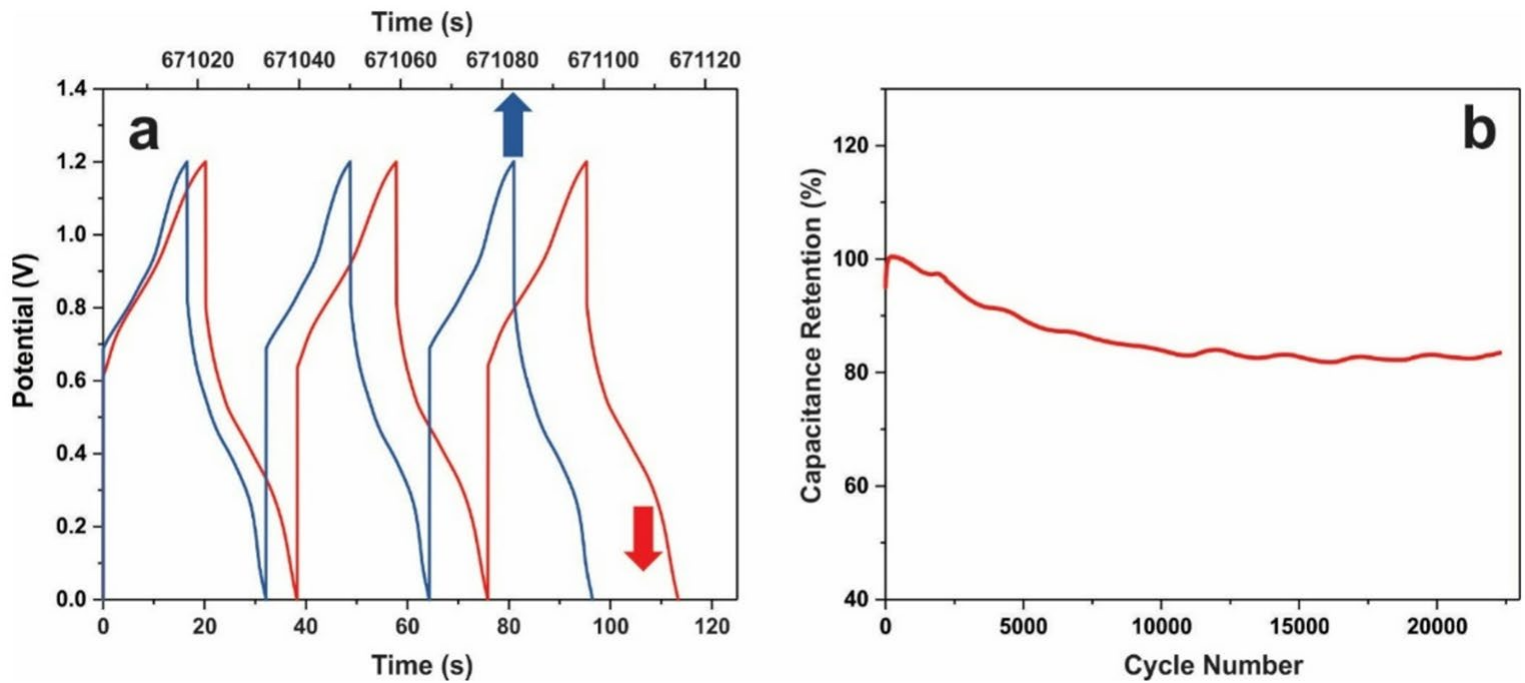
Cyclic performance of the Ni_3_S_2_/NF-AC/NF capacitor for 22,000 cycles: the first three GCD cycles and the last three cycles at a current of 450 mA **(a)**; capacitance retention vs. the number of cycles **(b)**.

**Fig. 9:**
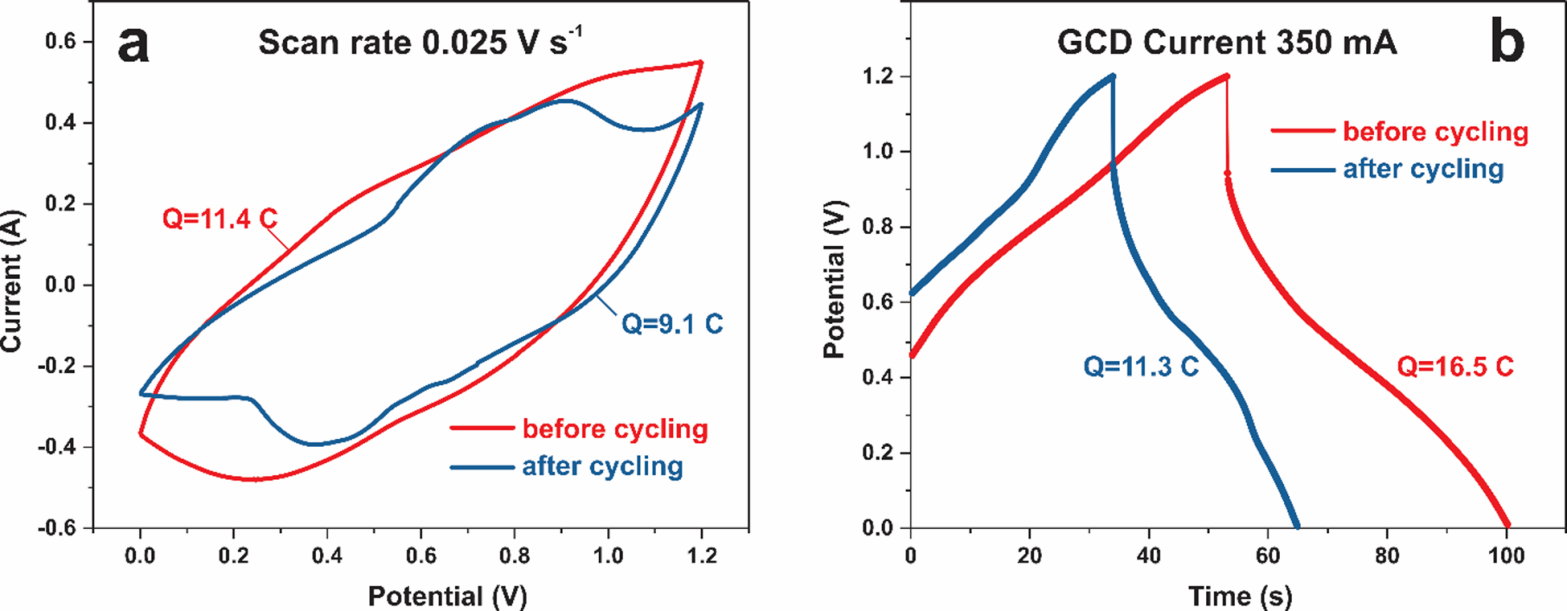
CV **(a)** and GCD **(b)** characteristics of Ni_3_S_2_/NF-AC/NF capacitor before and after 22,000 GCD cycles.

The assembled supercapacitor exhibited an areal capacitance of 3.85 C cm^–2^ (3.2 F cm^–2^) when measured by CV at a scan rate of 3 mV s^–1^. Using the GCD method, the areal capacitance was 4.64 C cm^–2^ (3.54 F cm^–2^) at a current density of 4 mA cm^–2^, decreasing from 100% to 65% as the current density increased from 4 to 250 mA cm^–2^. The cycling stability of the device was evaluated within a voltage window of 0–1.2 V at a GCD current of 450 mA over 22,000 cycles (Fig. 8). After approximately 10,000 cycles, the capacitance loss stabilized, with no further decrease observed during the subsequent 10,000 cycles. Minor fluctuations in the cycling curve are likely attributable to daily temperature variations of the electrochemical cell. Overall, the asymmetric supercapacitor demonstrated excellent cycling stability, retaining approximately 83% of its initial capacitance. A comparison of the CV and GCD characteristics before and after cycling is shown in Fig. 9a and b.

## Discussion

In this work, a simple and widely used sulfidation method was employed to fabricate a supercapacitor electrode by converting a porous nickel substrate into nickel sulfide. Owing to the robust structural support provided by nickel foam, this approach is well suited for practical applications. The resulting Ni_3_S_2_-based electrode exhibited an areal capacitance of approximately 4 C cm^–2^, which is typical for electrodes synthesized using this method. However, it is important to emphasize an aspect of Ni_3_S_2_-based electrodes that has thus far received limited attention. Unlike many other battery-type materials, which undergo the expected and irreversible degradation of capacity upon prolonged cycling, Ni_3_S_2_ electrodes often display unusually high resistance to cycling degradation. In some cases, the electrode capacitance even increases during extended galvanostatic charge–discharge (GCD) cycling, reaching 10,000–30,000 cycles. The magnitude of this enhancement can range from modest to highly pronounced, depending on the cycling conditions. For example, Ref^49^. reported an almost 88-fold increase in the initial capacitance of a Ni_3_S_2_ electrode after 20,000 charge–discharge cycles. This dramatic electrochemical activation of the as-synthesized electrode was attributed to the in situ formation of a porous Ni(OH)_2_ layer on the Ni_3_S_2_ surface. The emergence of the Ni(OH)_2_ phase was evidenced by the appearance of broad Raman bands with maxima at 460 and 536 cm^–1^, accompanied by the disappearance of Raman features associated with Ni_3_S_2_. Elemental mapping further revealed a decrease in sulfur content and a predominance of Ni and O in the subsurface region, indicating substantial surface restructuring.

Although numerous studies have investigated the sulfidation of porous nickel substrates for operation in alkaline electrolytes, the specific role of an in situ–formed porous Ni(OH)_2_ layer on Ni_3_S_2_ surfaces has received comparatively little attention. This represents an important yet underexplored mechanism that may largely account for the unusual capacitance enhancement observed in Ni_3_S_2_-based electrodes. Our results demonstrate that the in situ formation of Ni(OH)_2_ layers on the surface of Ni_3_S_2_, resulting in Ni(OH)_2_/Ni_3_S_2_/NF heterostructures, exerts a decisive influence on the electrochemical properties of the electrode.

Already at the early stages of electrochemical cycling (Fig. 4), Ni_3_S_2_ undergoes electrochemical activation through redox reactions involving the Ni^2+^/Ni^3+^ transition. These reactions lead to the release of sulfur species and the formation of an oxidized surface layer, as evidenced by the disappearance of the spectroscopic features characteristic of the Ni_3_S_2_ phase. This oxidized pseudocapacitive layer, formed on the surface of Ni_3_S_2_ during the initial stages of cycling, is extremely thin and therefore not detectable by X-ray diffraction.

Nevertheless, Raman spectroscopy clearly reveals structural rearrangements at early stages of electrochemical cycling (Fig. 2b): the characteristic Raman bands of Ni_3_S_2_ vanish after approximately 50 CV cycles within the potential range of − 0.7 to + 0.9 V vs. Ag/AgCl, while new bands attributable to nickel oxide and oxyhydroxide species emerge. Thus, the electrochemical performance of Ni_3_S_2_/NF electrodes is governed not by the pristine sulfide itself, but by its rapid transformation into an electrochemically active Ni–O/Ni(OH)_2_ surface layer during relatively short cycling periods (fewer than 100 cycles). Prolonged GCD cycling in an alkaline medium within a potential window of 0–0.45 V vs. Ag/AgCl can further promote this transformation through the following reaction:

3 $${\mathrm{N}}{{\mathrm{i}}_3}{{\mathrm{S}}_2}\,+\,6{\mathrm{O}}{{\mathrm{H}}^ - } \to 3{\mathrm{Ni}}{\left( {{\mathrm{OH}}} \right)_2}+{\text{ }}2{{\mathrm{S}}^2}^{-}.$$

Because this potential window (0–0.45 V vs. Ag/AgCl) coincides with the Ni^2+^/Ni^3+^ redox transition, long-term cycling promotes the gradual conversion of the sulfide phase into hydroxide without inducing excessive oxidation. As cycling proceeds, the thickness of the resulting hydroxide layer increases, eventually becoming detectable by X-ray diffraction (XRD) (Fig. 2a). The emerging nanocrystalline layer, which is structurally similar to β-Ni(OH)_2_, is largely electrochemically inactive under these conditions. First, the upper potential limit of 0.45 V is insufficient to drive the redox reaction

4 $${\mathrm{Ni}}{\left( {{\mathrm{OH}}} \right)_2}+{\text{ O}}{{\mathrm{H}}^ - } \leftrightarrow {\mathrm{NiOOH}}\,+\,{{\mathrm{H}}_2}{\mathrm{O}}\,+\,{{\mathrm{e}}^ - }.$$

throughout the full thickness of the growing layer. Second, the β-phase possesses a densely packed structure that restricts the intercalation of OH^–^ ions. In addition, the increasing electrical resistance of the thickening hydroxide layer may further contribute to the observed decrease in capacitance during prolonged cycling.

The present study also reveals a capacitance activation phenomenon (Fig. 6), whereby capacitance lost during GCD cycling can be partially recovered through subsequent electrochemical activation. Increasing the electrode potential to 1.0–1.5 V vs. Ag/AgCl induces deep oxidation and hydration of β-Ni(OH)_2_, in accordance with the reaction

5 $$\beta - {\mathrm{Ni}}{\left( {{\mathrm{OH}}} \right)_2} + {\text{ }}{{\mathrm{H}}_2}{\mathrm{O}} \to \alpha - {\mathrm{Ni}}{\left( {{\mathrm{OH}}} \right)_2}{{\mathrm{H}}_2}{\mathrm{O}},$$

leading to the formation of α-Ni(OH)_2_-like structures (Fig. 2a, curve 4). The phases are characterized by an open-layered morphology and enhanced capacitance^20^. These transformations account for the exceptionally high capacitance and excellent cycling stability observed in the present system. The newly formed hydroxide layer exhibits a highly dispersed nanocrystalline morphology, as evidenced by the XRD results. Owing to its highly active surface and in situ formation, the nanocrystalline Ni(OH)_2_ layer provides a substantially larger number of electrochemically active sites, thereby compensating for its relatively lower theoretical specific capacity.

Importantly, during electrochemical hydration, the morphology of the hydroxide layer cannot develop electrochemically inaccessible regions, as the presence of electrolyte is a prerequisite for the in situ formation of Ni(OH)_2_. Consequently, the resulting Ni(OH)_2_ layer exhibits improved electrolyte accessibility and more efficient electrochemical utilization compared with bulk Ni_3_S_2_.

Although nickel hydroxides exhibit higher electrical resistance than Ni_3_S_2_, electrochemical hydration promotes the formation of a hydroxide layer that is free of regions with severely hindered electron transport. Consequently, the increase in electrode resistance is far less pronounced than would be expected if the layer were produced by alternative methods, such as chemical or thermal oxidation. Moreover, the thin hydrated layer formed on the surface of Ni_3_S_2_ acts as a mechanical buffer, accommodating volume changes and associated stresses, thereby enhancing the structural stability of the electrode during prolonged electrochemical cycling.

As shown in Fig. 7, following electrochemical activation, the potential separation between the redox peaks in the cyclic voltammetry (CV) curves increases relative to that of the pristine electrode. This behavior suggests that the redox-active centers are located deeper within the material, and that under kinetic limitations there is insufficient time for their full participation during galvanostatic charge–discharge (GCD) measurements at high current densities.

Because Ni_3_S_2_/NF supercapacitor electrodes—widely studied in the literature—are almost invariably evaluated in alkaline electrolytes, it is essential to account for the formation of composite electrode architectures consisting of Ni(OH)_2_ surface layers on a Ni_3_S_2_/NF framework when interpreting their electrochemical properties and predicting device performance. Although such composites benefit from the synergistic combination of a highly active nanocrystalline hydroxide layer and a highly conductive Ni_3_S_2_/NF backbone, excessive growth of the hydroxide layer can introduce transport limitations and increase internal resistance. Therefore, precise control over hydroxide layer formation is critical for preserving electrode structural integrity and achieving optimal electrochemical performance in supercapacitor applications. Maintaining a delicate balance between the conductive sulfide backbone and the in situ–formed hydroxide layer is essential to maximize the density of electrochemically active sites while minimizing charge-transport resistance.

## Conclusions

This study of Ni_3_S_2_ electrodes grown on nickel foam via hydrothermal sulfidation demonstrates that electrode capacitance increases significantly under electrochemical operation in alkaline electrolytes. The electrodes exhibit high stability of their electrochemical parameters during prolonged galvanostatic charge–discharge (GCD) cycling, as well as a pronounced capacitance recovery upon activation in cyclic voltammetry (CV) mode. These unusual behaviors originate from the in situ formation of a nanocrystalline hydroxide layer on the surface of the Ni_3_S_2_/NF electrode. The oxidized surface layer forms during the early stages of electrochemical redox cycling, leading to a rapid increase in capacitance and a substantial restructuring of the electrode surface, accompanied by the disappearance of spectroscopic features characteristic of the Ni_3_S_2_ phase. The formation of a β-Ni(OH)_2_/Ni_3_S_2_/NF heterostructure is unequivocally confirmed by Raman spectroscopy, X-ray diffraction (XRD), and X-ray photoelectron spectroscopy (XPS). Subsequent emergence of the α-Ni(OH)_2_ phase following CV activation indicates rapid phase transformations within the nanocrystalline oxidized layer under electrochemical conditions.

The thin hydroxide layer is primarily responsible for the high specific capacitance, while the underlying Ni_3_S_2_ layer ensures high electrical conductivity and provides strong mechanical and electrical integration with the nickel foam substrate. The synergistic performance of the Ni(OH)_2_/Ni_3_S_2_/NF heterostructure arises from the optimized thickness of the hydroxide layer, which is governed by in situ electrochemical oxidation of Ni_3_S_2_. This process yields a nanocrystalline hydroxide layer with a highly accessible morphology, thereby maximizing the electrochemically active surface area available to the electrolyte.

Because the propensity for electrochemical oxidation is a general characteristic of metal sulfides, the formation of near-surface hydroxide layers must be carefully considered when employing sulfide-based electrodes in alkaline electrolytes. Controlled in situ surface oxidation of metal sulfide electrodes to form thin hydroxide layers therefore represents a rational strategy for the development of sulfide-based heterostructures and for enhancing their electrochemical performance.

## Supplementary Information

Below is the link to the electronic supplementary material.

Supplementary Material 1

## Data Availability

All data generated or analysed during this study are included in this published article.
