# Supplementary material for: In situ formation of nanocrystalline Ni(OH)2 in alkaline electrolyte explains superior capacitance and cycling stability of Ni3S2/NF electrodes
Source: Sci Rep. 2026 Mar 5;16:12209. doi: 10.1038/s41598-026-42576-y (PMC13076998; doi:10.1038/s41598-026-42576-y)
Supplement: Supplementary file 1 — Supplementary Material 1 [file 41598_2026_42576_MOESM1_ESM.docx]

**In Situ Formation of Nanocrystalline Ni(OH)_2_ in Alkaline Electrolyte Explains Superior Capacitance and Cycling Stability of Ni_3_S_2_/NF Electrodes**

Kh.A. Abdullin^1^, M.T. Gabdullin^2^, L.V. Gritsenko^1,3*^, Zh.K. Kalkozova^1^, Zh.S. Kanatov^1^,

A.А. Markhabayeva^1^, R.R. Nemkayeva^1^, D. Zhapargali^1,4^, M. Mirzaeian^5^

^1^ National Nanotechnology Laboratory of Open Type of Al-Farabi Kazakh National University, al-Farabi ave., 71, Almaty, 050040, Kazakhstan

^2^ School of Materials Science and Green Technology, Kazakh-British Technical University, Tole bi Street, 59, Almaty, 050000, Kazakhstan

^3^ Satbayev University, General Physics Department, Satpayev str., 22, Almaty, 050013, Kazakhstan

^4^ Satbayev University, Department of Materials Science, Nanotechnology and Engineering Physics, Satpayev str., 22, Almaty, 050013, Kazakhstan

^5^ School of Computing, Engineering and Physical Sciences, University of the West of Scotland, Paisley, PA1 2BE, United Kingdom

* Correspondence: l.gritsenko@satbayev.university


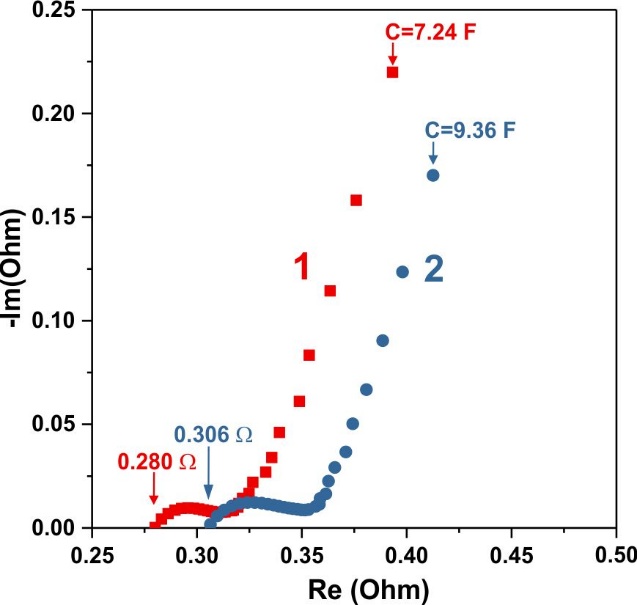


**Figure S1**. Nyquist plot for Ni_3_S_2_/NF electrode area of 4 cm^2^: before GCD cycling (red squares); and after 10,000 GCD cycles in the potential range 0-0.45 V vs. Ag/AgCl, followed by CV activation up to 1.5 V (blue circles). The bias voltage of 0.4 V vs. Ag/AgCl, frequency range from 50 kHz to 0.1 Hz.


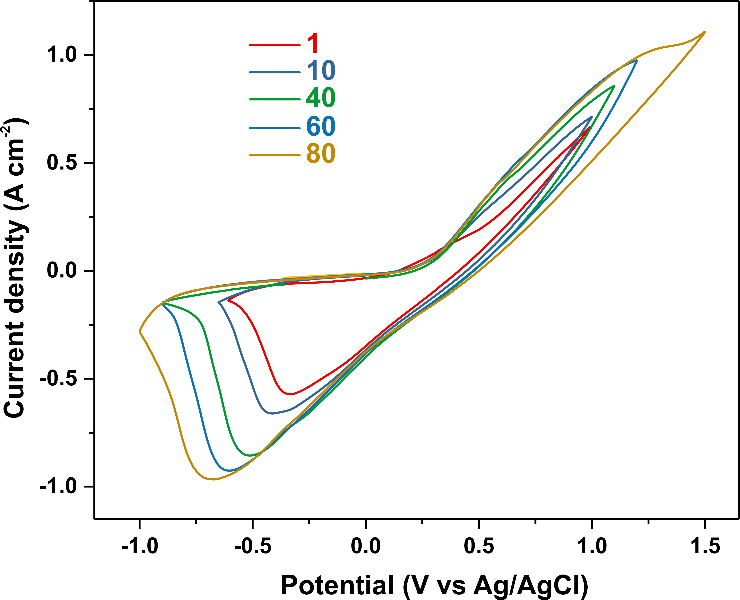


**Figure S2.** CV curves showing capacitance recovery of the Ni_3_S_2_/NF electrode after 10,000 GCD cycles. The numbers on the curves denote the number of CV cycles. The scan rate was 0.1 V s^–1^.

| 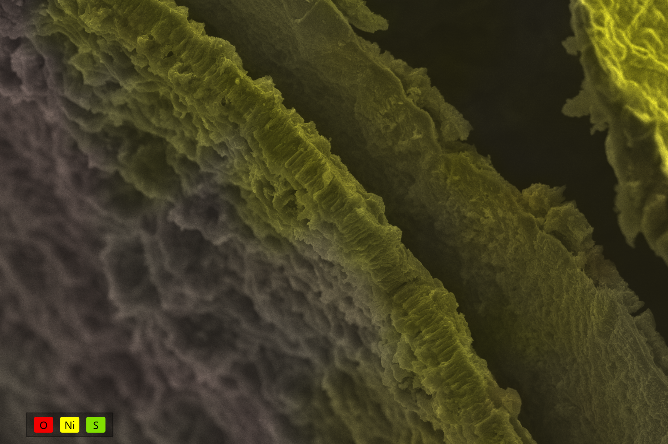 | 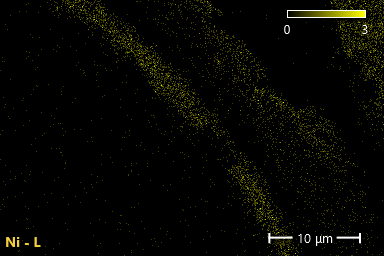 |
| --- | --- |
| a | b |
| 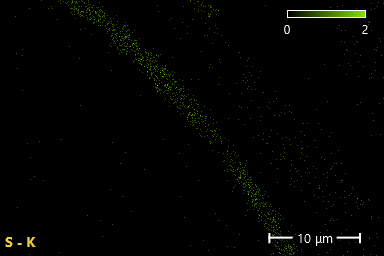 | 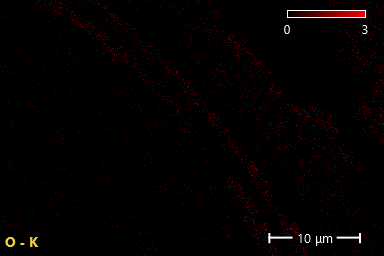 |
| c | d |
| 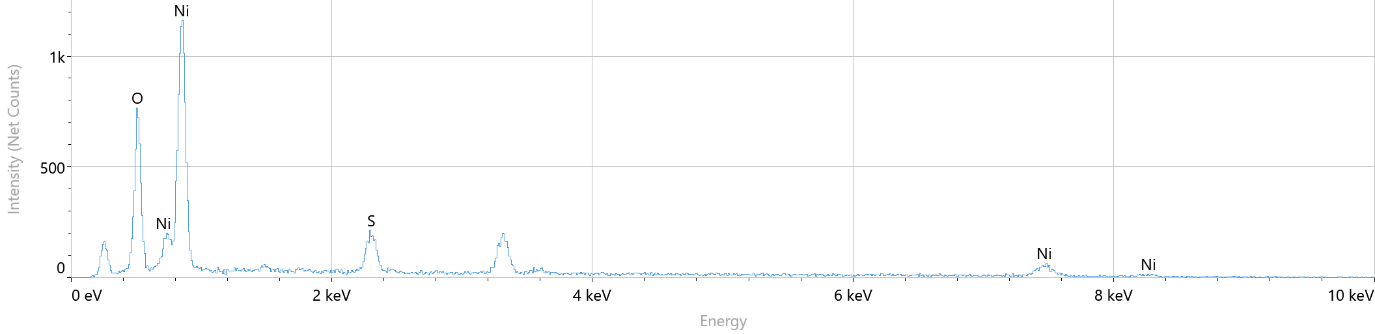 | |
|  | |

| Element | Line | At. % | Wt. % | Net Counts | At. % Error | Wt. % Error |
| --- | --- | --- | --- | --- | --- | --- |
| O | K | 50.1 | 23.1 | 4 363 | 0.6 | 0.3 |
| S | K | 9.8 | 9.1 | 2 107 | 0.4 | 0.3 |
| Ni | L | 40.1 | 67.8 | 10 211 | 0.4 | 0.7 |

**Figure S3.** SEM elemental mapping of the sample prior to galvanostatic charge–discharge (GCD) cycling. Panels (a–d) show, respectively, a cross-sectional overview of the Ni₃S₂ film on the nickel foam (NF) substrate and the corresponding spatial distributions of nickel, sulfur, and oxygen. The energy-dispersive X-ray (EDX) spectrum, acquired at an accelerating voltage of 10 keV, along with a table summarizing the elemental composition, is also presented.

| 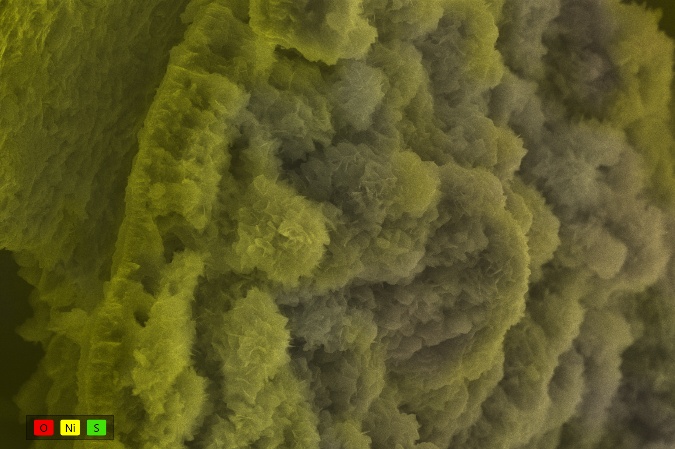 | 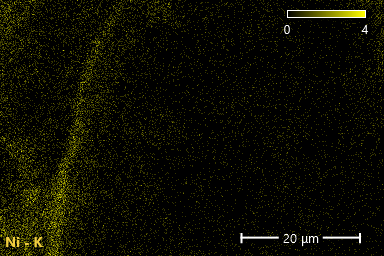 |
| --- | --- |
| a | b |
| 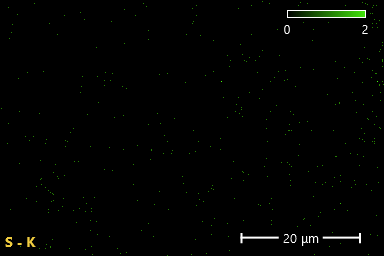 | 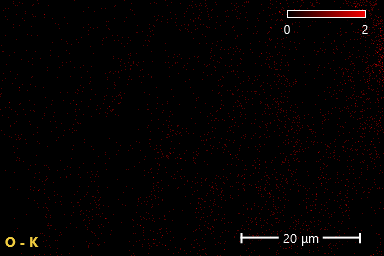 |
| c | d |
| 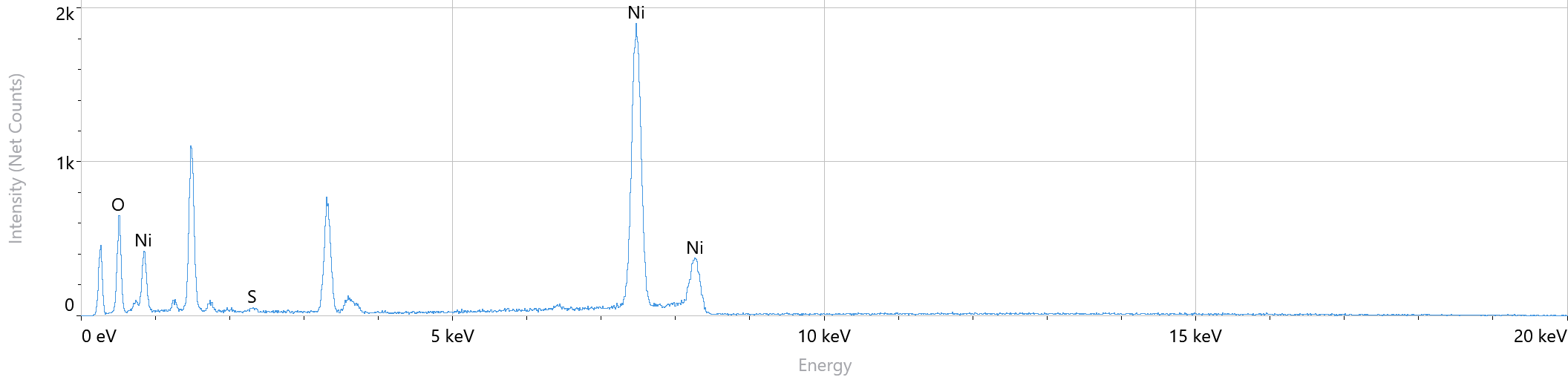 | |
|  | |

| Element | Line | At. % | Wt. % | Net Counts | At. % Error | Wt. % Error |
| --- | --- | --- | --- | --- | --- | --- |
| O | K | 30.1 | 10.5 | 4 198 | 0.5 | 0.2 |
| S | K | 0.5 | 0.3 | 400 | 0.1 | 0.0 |
| Ni | K | 69.4 | 89.2 | 32 004 | 0.7 | 1.0 |

**Figure S4.** SEM elemental mapping of the sample after 30,000 GCD cycles. Panels (a–d) present, respectively, a cross-sectional overview of the Ni₃S₂ film on the NF substrate and the corresponding spatial distributions of nickel, sulfur, and oxygen. The EDX spectrum, acquired at an accelerating voltage of 20 keV, together with a table summarizing the elemental composition, is also shown.


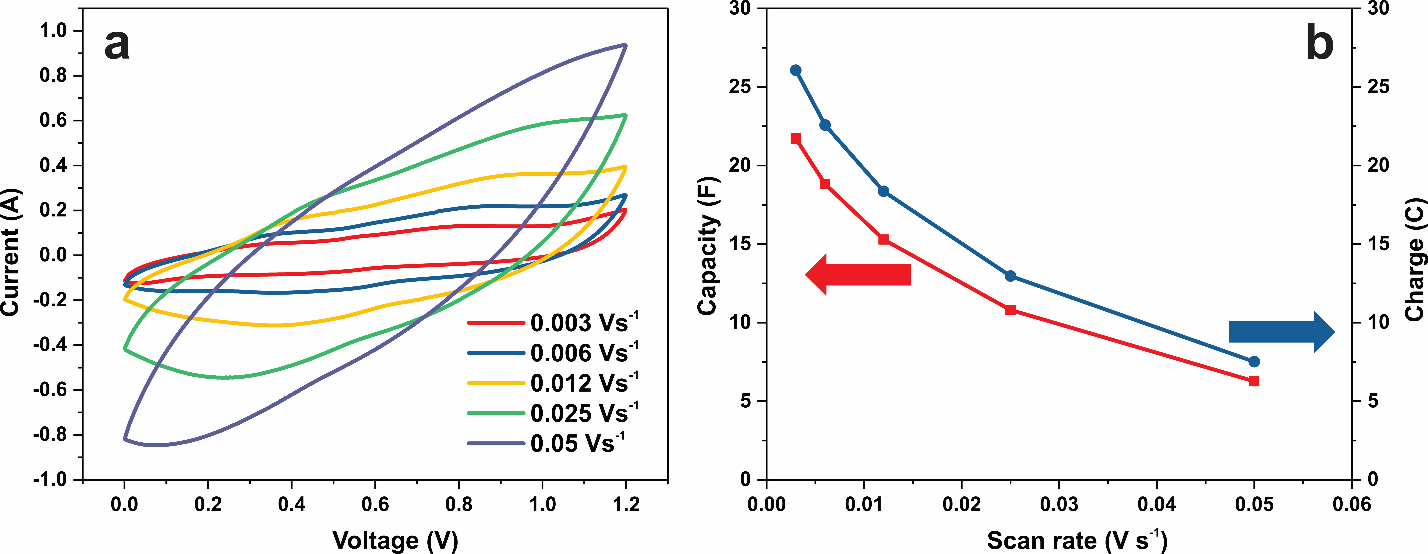


**Figure S5.** The CV curves for the Ni_3_S_2_/NF-AC/NF capacitor with an Ni_3_S_2_/NF electrode area of 6 cm^2^ at different scan rate (a) and the capacitance and charge of the capacitor vs. scan rate (b).


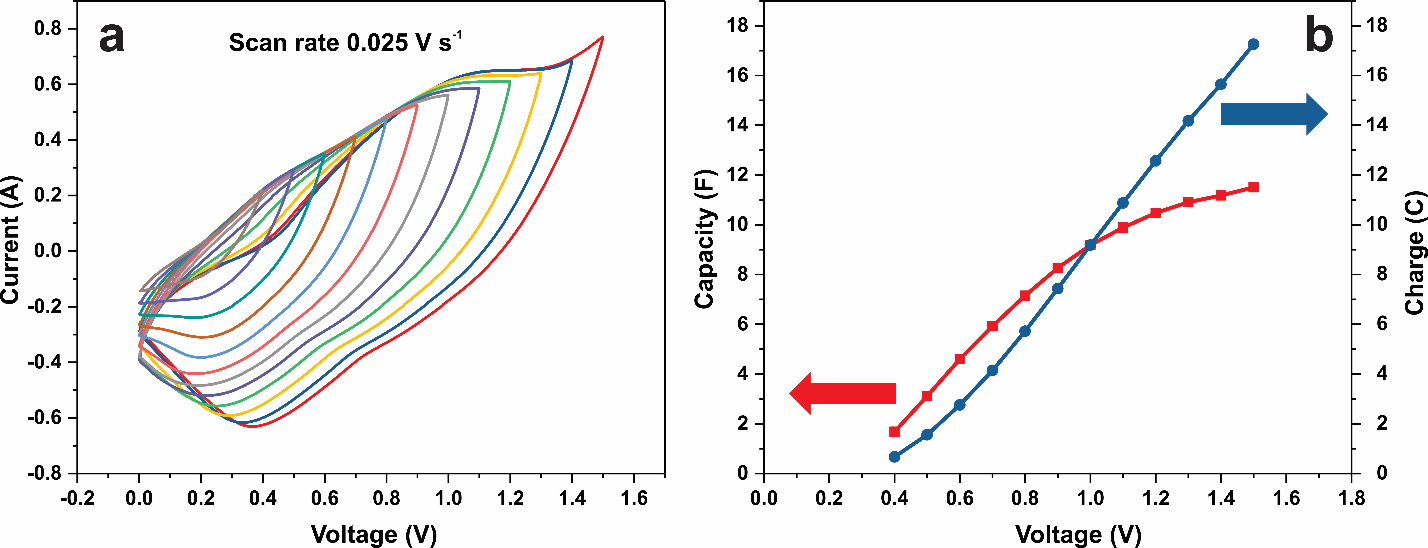


**Figure S6**. Cyclic voltammetry (CV) curves for the Ni_3_S_2_/NF-AC/NF capacitor at various potentials are shown in (a), while the capacitance and charge of the capacitor as a function of potential are presented in (b).


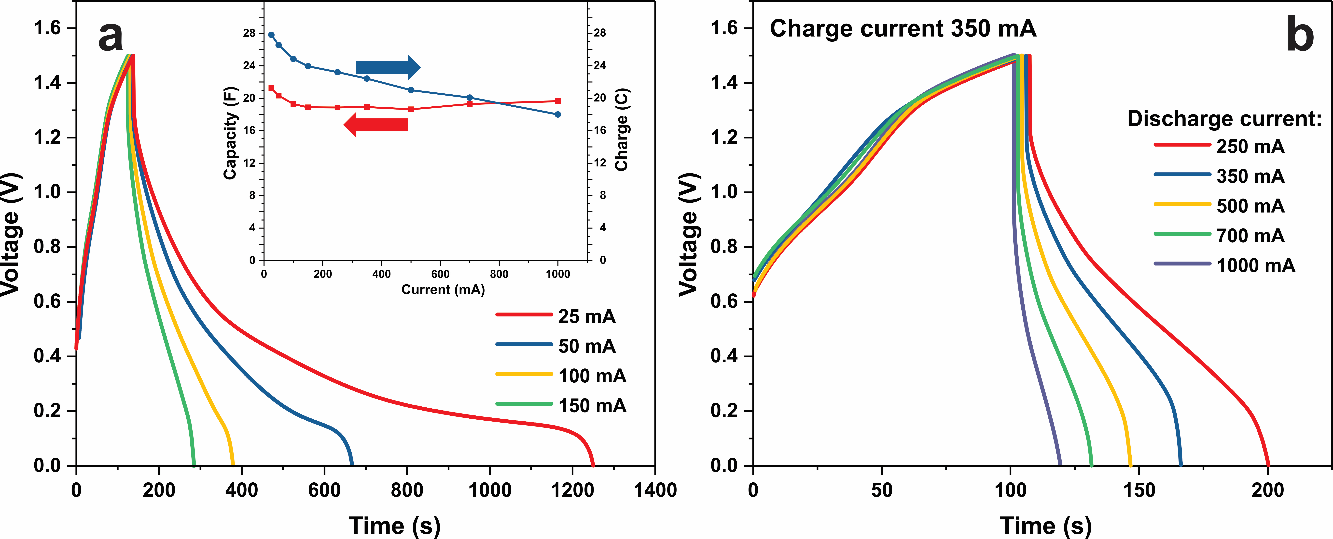


**Figure S7.** Charge-discharge (GCD) curves for the capacitor at a charging current of 350 mA and various discharge currents are presented. The inset illustrates the dependence of the capacitor's capacitance and charge on the discharge current.


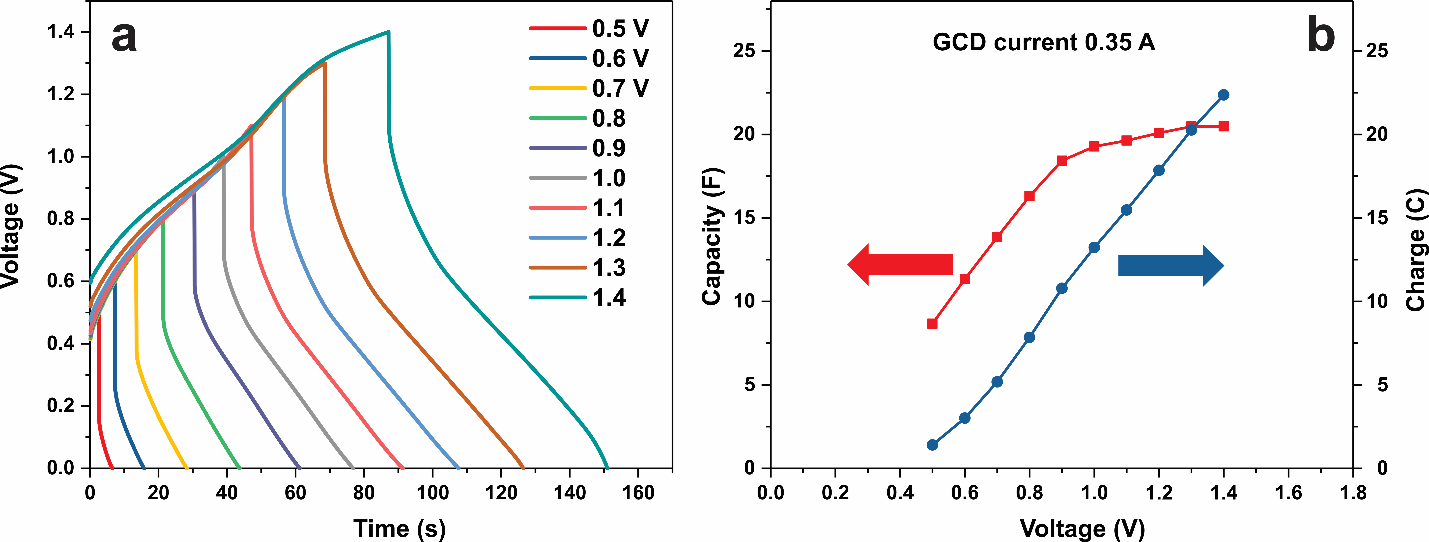


**Figure S8**. Charge-discharge curves for the capacitor at a charging current of 350 mA across various potentials are shown in (a), with the dependence of the capacitor's capacitance and charge on potential presented in (b).
